# Editorial: Identification of phenotypically important genomic variants

**DOI:** 10.3389/fbinf.2023.1328945

**Published:** 2023-11-10

**Authors:** Elizabeth A. Heron, Giorgio Valle, Anna Bernasconi

**Affiliations:** ^1^ Department of Psychiatry, Trinity College Dublin, Dublin, Ireland; ^2^ Department of Biology, Università di Padova, Padova, Italy; ^3^ Department of Electronics, Information, and Bioengineering, Politecnico di Milano, Milan, Italy

**Keywords:** next-generation sequencing (NGS), genomic variant, prioritisation, interpretation, annotation

Due to significant technological advances and reductions in cost, we are witnessing a rapid increase in Next-Generation Sequencing (NGS) studies including whole exome sequencing, whole genome sequencing, and RNA-seq (transcriptome) together with multi-omics approaches, in all areas of health, disease and research. NGS is not limited to human genomics, but is also having major impacts in the fields of plant, animal and pathogen research. However, these technologies present a significant challenge given the huge number of variants that are being identified and that require interpretation. This can make harnessing the potential of NGS difficult when it comes to analysing this data.

Due to the large amount of data, a frequently employed approach in NGS studies is to identify a manageable subset of genomic variations that can be used to further the understanding of the biological underpinnings of the phenotypes of interest. This list is obtained through variant filtering and prioritisation. Variant filtering aims to identify high-quality variant calls, removing false positives, with variant prioritisation aiming to identify phenotype-associated or causal variants. For both the variant filtering and prioritisation steps, several public and/or private annotation databases are typically consulted in a single study. Additionally, several tools have been developed (Anderson and Lassmann, 2022).

This Research Topic addresses the challenge of integrating the vast array of annotation resources available in the filtering and prioritisation steps of NGS studies, taking into account the diversity of potential applications, ranging from routine diagnosis of genetic diseases to prioritisation of novel variants in genes with unknown functions. In the clinical context, the American College of Medical Genetics and Genomics and the Association for Molecular Pathology (ACMG/AMP) guidelines (Richards et al., 2015) have been developed. However, these still present challenges (Vihinen, 2020). Computational, artificial intelligence, machine learning, statistical modelling and simpler approaches can all offer potential solutions for this integration depending on the context.

Three papers included in this Research Topic have tackled the variant identification effort in the context of human genomics, whereas one paper addresses pathogen variants.

In *Assessing the pathogenicity of BRCA1/2 variants of unknown significance: Relevance and challenges for breast cancer precision medicine*, De Paolis et al. describe in great detail the reasoning applied to assign pathogenicity evidence to a variant of uncertain significance (VUS) of the BRCA1 gene. The variant was found in a 65 year old woman affected by breast cancer, in her 35 year old daughter affected by adenocarcinoma of the uterine cervix and in a 41 year old niece also affected by breast cancer. Three levels of analysis were performed: 1) association of the BRCA1 variant to cancer-affected members; 2) absence of other high-risk mutation; 3) multiple indirect evidence derived from gene and protein structural analysis. Each level of the analysis is thoroughly covered in the paper. In general, VUSs cannot be classified as clinically relevant, due to insufficient evidence. This study underlines the need for a dedicated clinical path for patient carriers of a VUS, in order to evaluate the significance of the VUS and to speculate about its classification.

In *Protein domains provide a new layer of information for classifying human variations in rare diseases*, Corcuff et al. propose a method to address the challenge in sequence variant interpretation presented by the ACMG/AMP PM1 criterion associated with protein domains which is only assigned in about 10% of cases. The authors present DOLPHIN which uses Pfam alignments of eukaryote sequences to develop a scoring system to identify protein domain residues and variants that have a significant impact. The authors go on to validate these new scores using ClinVar data. Using this method of scoring, 30% of potential human transcripts’ variants could be assigned the PM1 criterion and the authors propose a new benign support criterion, BP8. DOLPHIN scores also provide a method of extrapolating variant frequencies which could be applied to 31.8% of variants, in comparison to the use of gnomAD where frequency information was only available for 7.6% of variants.

Sharing of raw data and adhering to the Findability, Accessibility, Interoperability, and Reusability (FAIR) data principles, ensures rapid reproducibility and the possibility to progress technique development, in particular bioinformatics techniques, to further our understanding of sequence variation. Data repositories such as the European Genome-phenome Archive (EGA) facilitate the storage of encrypted data and regulate access to the data. However, the process to deposit data and ensure the minimisation of errors is time-consuming (estimated to take 1 month). In *EGAsubmitter: A software to automate submission of nucleic acid sequencing data to the European Genome-phenome Archive*, Viviani et al. have provided a pipeline to guide users in all steps (from file encryption to upload to metadata submission) of the submission process of sequencing data to EGA. This pipeline is expected to streamline the process of automated submission and minimise errors.

The labeling system for SARS-CoV-2 proposed by the World Health Organization and adapted by the United States Centers for Disease Control and Prevention (CDC) is based on the virus’ six major attributes (including transmissibility, disease presentation, and epidemiological impact), from which 24 criteria are derived.

In a pandemic context, the necessity of rapidly labeling a new variant and the lack of scientific evidence presents unclear criteria. As these criteria are formulated differently by each agency, there are discrepancies in the labels of currently active variants, with consequences also for data applications that identify (Bernasconi et al., 2021) and hunt (Pinoli et al., 2023) SARS-CoV-2 emerging variants. In *How concerning is a SARS-CoV-2 variant of concern? Computational predictions and the variants labeling system*, Ashoor et al. evaluate the in-use labeling system and propose a predictive computational comparative approach for rapid and accurate labeling of SARS-CoV-2 variants, by finally providing a harmonization of the labeling system useful for data integration efforts (Alfonsi et al., 2022) and the wider research community.

In essence, this Research Topic covers the exciting developments in the approaches for the integration of annotation resources for variant filtering and prioritisation. It will be a valuable resource for computational biologists, bioinformaticians, clinicians, and researchers working in the field of genetic variation.
